# Complete genome sequencing and comparative genomic analyses of a new spotted-fever *Rickettsia heilongjiangensis* strain B8

**DOI:** 10.1080/22221751.2022.2153085

**Published:** 2023-02-13

**Authors:** Maozhang He, Lifeng Zhang, Haoran Hu, Xiaohan Liu, Cong Zhang, Yu Xin, Boyu Liu, Zhen Chen, Kehan Xu, Yan Liu

**Affiliations:** Department of Microbiology, School of Basic Medical; The Key Laboratory of Microbiology and Parasitology of Anhui Province, The Key Laboratory of Zoonoses of High Institutions in Anhui, Anhui Medical University, Hefei, People’s Republic of China

**Keywords:** *Rickettsia heilongjiangensis*, spotted fever group rickettsia, whole-genome sequencing, comparative genomic analyses, functional pathways

## Abstract

*Rickettsia heilongjiangensis*, a tick-borne obligate intracellular bacterium and causative agent of spotted fever in China, has attracted increasing concern regarding its capability in causing human rickettsiosis. Here, we conducted a genomic analysis of a new *R. heilongjiangensis* strain B8 (B8) isolated from the serum of a patient who had been bitten by a *Haemaphysalis longicornis* tick in Anhui Province, China. The present study sought to identify exclusive genes that might be associated with the pathogenicity of B8 using comparative genomics. Specifically, the sequences of B8 were assembled into one circular chromosome of 1,275,081 bp and predicted to contain 1447 genes. Comparative genome analyses were performed based on the genome of B8 and 28 spotted fever group (SFG) rickettsial genomes deposited in NCBI. Phylogenomic analyses indicated the B8 strain was clustered within the *R. heilongjiangensis* species; however, a sum of 112 and 119 B8-unique genes was identified when compared with *R. heilongjiangensis* and *R. japonica* strains, respectively. Functional annotation analyses revealed that these B8-unique genes were mainly annotated to defence mechanisms, lipid transport and metabolism, cell wall/membrane/envelope biogenesis. These data indicate B8 rather represents a previously undescribed human-pathogenic SFG rickettsia lineage, which may be an intermediate lineage of *R. heilongjiangensis* and *R. japonica*. Overall, this study isolated a new strain of *R. heilongjiangensis* in East-Central China for the first time, and provided potential B8-unique genetic loci that could be used for the discrimination of B8 from other R. *heilongjiangensis* and closely related SFG *Rickettsial* strains.

## Introduction

Spotted Fever Group of rickettsiae (SFGR) are a neglected and understudied group of exclusively intracellular pathogens belonging to the genus *Rickettsia*, and are agents of a large number of emerging infectious pathogens with a worldwide distribution [1,2]. SFGR causes diseases called spotted fever, which is zoonotic and is mainly transmitted to the host by the bite of ticks, posing a serious threat to human health. Once SFGRs invade the host, it primarily targets and multiplies in small vascular endothelial cells and reticulocyte system, leading to increased vascular permeability [3], causing vascular endothelial damage and inflammation. This insult to the vasculature leads to oedema of the tissue around the lesion and reduced blood volume of the local microcirculation, resulting in vasculitis, perivascular inflammation, and organ lesions. Patients develop rash, ulceration, fever, local lymph node enlargement, skin bruising necrosis, and finger gangrene [4,5]. Disease progression can be fatal with reported complications, such as hypotensive shock, acute renal failure, pulmonary oedema, meningitis, and vital organ dysfunction [6].

*Rickettsia heilongjiangensis* is antigenically defined as spotted fever group rickettsia and causes a rickettsiosis termed Far-Eastern spotted fever (FESF) in humans. Strain 054, the type strain of *R. heilongjiangensis*, was first isolated from *Dermacentor silavrum* ticks in the Heilongjiang Province, China, in 1982 [7]. Previous research shown that the *R. heilongjiangensis* is originally classified as one of the subgroup of *R. japonica* [8]. Prior to this, the epidemic zone of *R. heilongjiangensis* was reported mainly in the northeastern of China [9]. However, it strongly required to identify whether *R. heilongjiangensis* is epidemic in other regions of China. Therefore, the novel SFG Rickettsia isolates acquired from patients or ticks in different geographic locations are highly important to be studied, especially for the investigation of genomic and functional information, evolutionary transitions, and interaction between the host and *R. heilongjiangensis*.

In the present study, a newly isolated *R. heilongjiangensis* (B8) was sequenced and the molecular functions were investigated based on the whole-genome sequence (WGS). In addition, a comprehensive comparison among the genome of B8 and other SFG rickettsial strains was performed to better characterize the genomic divergences among these inter-/intra-species and to identify genetic traits and targets that can be used to distinguish the B8 strain from other SFG *Rickettsia* in the different geographic locations of China. The whole-genome sequencing and pan-genome analysis performed here were pivotal in inferring the evolutionary relationships among inter-/intra-*Rickettsia* species, contributing to the foci surveillance, and providing the ideal genome regions that can use as PCR targets of B8 to differentiate from other *R. heilongjiangensis* and related SFG *Rickettsia*.

## Materials and methods

### Isolation, purification, and genome extraction of *Rickettsia*

The B8 strain was isolated from human blood sample collected by the People’s Hospital of Hanshan County, Anhui province, central-eastern part of China. In order for propagation, the bacterium was cultivated in Vero cells (ATCC^®^ CCL-81™) and incubated in modified Eagle’s minimal essential medium supplemented with 4% foetal bovine serum and 2 mM L-glutamine at 34°C in an incubator with 5% CO_2_ for consecutive seven days as previously described [9,10]. The infected cells were disrupted by vortexing with glass beads, followed by centrifugation at 500 × *g* for 10 min to remove host cell debris. The supernatants were centrifuged at 10,000 × g for 30 min to recapture bacterial cells. The obtained pellets were resuspended in 1× PBS and then layered on 20–50% linear Renografin gradient and centrifuged at 197,000 × g. The rickettsial layer was collected and washed using 1× PBS for three times and then suspended in culture media. The viability at ∼95% of the purified bacteria was measured using a bacterial kit (LIVE/DEAD BacLight Bacterial Viability Kits, Invitrogen). The rickettsial genomic DNA extraction was extracted a QIAamp DNA minikit (Qiagen, Germany) according to manufacturer’s protocols, eluted in a final volume of 100 µl, and stored at −80°C until use.

### Ticks collection, DNA isolation, and PCR screening for positive *R. heilongjiangensis*

Ticks were collected by flagging over vegetation in the territory of Hanshan County near the patient lived, and the collection sites were either large forests or grasses near woodlands. The ticks were kept alive in 50 ml plastic tubes until identification and further testing in the laboratory. The ticks were firstly dipped in 75% ethanol two times (10 min for each time) and then washed twice in distilled water and air-dried prior to homogenization in 1.5 mL Eppendorf tubes with disposable pestles and subsequent DNA extraction using the QIAamp^®^ DNA Blood Mini Kit (Qiagen, Germany) following the manufacturer’s instruction [11,12]. *Rickettsia heilongjiangensis* was identified by PCR screening with four pairs premiers (Table 1).

**Table 1.** The information of public *Rickettsia* species (retrieved from NCBI).

Representative genome of *R*. sp.	Location	Year of isolation	Origin/Tick vector	Selected strain	Genome size (Mp)	Accession No.
*R. heilongjiangensis*	Sendai, Japan	2008	*Haemaphysalis concinna*	Sendai-29	1.28	AP019864
* *	Heilongjiang province, China	1983	*Dysmicoccus sylvarum*	54	1.28	CP002912
* *	Inner Mongolia, China	1996	*Haemaphysalis concinna*	CH8-1	1.28	AP019862
* *	Sendai city, Japan	2009	*Haemaphysalis concinna*	Sendai-58	1.28	AP019865
* *	Sendai city, Japan	2012	*Haemaphysalis concinna*	HCN-13	1.28	AP019863
*R. japonica*	Okayama, Japan	1985	Isolated from patient	YH	1.28	AP011533
* *	Miyazaki, Japan	2008	Isolated from patient	MZ08014	1.28	AP017577
* *	Kochi, Japan	1985	Isolated from patient	YH_M	1.28	AP017602
* *	Kochi, Japan	2004	Isolated from patient	Tsuneishi	1.28	AP017581
* *	Wakayama, Japan	1994	Isolated from patient	Nakase	1.28	AP017578
* *	Nagasaki, Japan	2007	Adult *Haemaphysalis hystricis*	HH07167	1.28	AP017576
* *	Tokushima, Japan	1993	Larva *Dermacentor taiwanensis*	DT-1	1.28	AP017572
* *	Ehime, Japan	2006	Adult *Haemaphysalis hystricis*	HH06154	1.28	AP017574
* *	Miyazaki, Japan	2007	Nymph *Haemaphysalis hystricis*	HH07124	1.28	AP017575
* *	Okayama, Japan	2011	Isolated from patient	PO-1	1.28	AP017580
* *	Kagoshima, Japan	2003	*Haemaphysalis hystricis*	HH-1	1.28	AP017573
* *	Zhejiang Province, China	2015	Isolated from patient	LA16/2015	1.28	CP047359
* *	Zhejiang Province, China	2015	Isolated from patient	LA4/2015	1.28	CP032049
* *	Okayama, Japan	2012	*Haemaphysalis hystricis*	OHH-1	1.28	AP017579
*R. akari*	/	/	Isolated from patient	Hartford	1.23	CP000847
*R. slovaca*	Slovakia	1968	*Dermacentor marginatus*/*Dermacentor reticulatus*	13-B	1.28	CP003311
*R. philipii*	/	/	/	TH1527	1.29	CP003308
*R. montanensis*	/	/	/	OSU 85-930	1.28	CP003340
*R. parkeri*	Necoclí, Colombia	2010	*Amblyomma ovale*	Atlantic Rainforest	1.35	CP040325
*R. raoultii*	Russia	2005	*Dermacentor silvarum*	Khabarovsk	1.34	CP010969
*R. rickettsii*	USA	1938	*Dermacentor variabilis*	Iowa	1.27	CP018913
*R. montanensis*	Montana, USA	1963	Dermacentor Variabilis/Dermacentor Andersoni	OSU 85-930	1.28	CP003340
*R. sibirica*	Krasnojarsk, Russia	1949	/	246	1.25	AABW01000001

### Whole-genome sequencing, assembly, and annotation of B8

The B8 strain isolated in this study was subjected for next-generation sequencing by using the Nextera XT DNA Sample Preparation kit (Illumina, San Diego, CA, USA) and the Illumina MiSeq platform. After filtering adaptors and low-quality reads by Fastp (version 0.20.1) using default settings [13], then we performed *de novo* assembly of the genome sequence by using SPAdes v 3.12.0 as previously described [14]. The taxonomic information of this genome sequence was identified by using Kraken2 against RefSeq annotation based on *k-mers* strategy. The genome sequence is available at the NCBI’s GenBank under the accession ID: CP112971.

The circular map of the genome was generated and represented using CGView server (http://cgview.ca/) [15]. Protein-coding regions in the assembled sequences were predicted using Prodigal [16]. In addition, the estimation of the number of tRNA and rRNA sequences were identified by tRNA-scan and RNAmmer, respectively. The genomes of B8 and other publicly downloaded SFGRs were annotated by a command line software: Prokka (v 1.14.1) [17]. For functional annotation, the genome sequences were examined using several protein databases. In brief, the function of predicted protein CDSs was determined against the NCBI non-redundant (NR), Gene Ontology (GO), eggnog 5.0 [18], and Kyoto Encyclopedia of Genes and Genomes (KEGG) databases with E-value cut-off set to 1e−5 and subsequent filtering for the best hit. In addition, the whole genome was blasted against VFDB and CARD for screening virulence genes and resistance genes with ABRicate (https://github.com/tseemann/abricate).

Prophage regions were predicted, and figures were generated using PHAST (http://phast.wishartlab.com) [19]. As submitted to NCBI, the whole chromosome was uploaded to analysis using PHAST to predict the completeness of the prophage regions, and to identify and map their elements. Insertion sequence (IS) elements were predicted using IS Finder software (http://issaga.biotoul.fr/issaga_index.php) [20]. Genomic islands were predicted using IslandPath and SIGI-HMM, part of software IslandViewer4 [21], to identify regions with evidence of horizontal transfer in that of the B8 genome. Predicted surface proteins were carried out using Signal-P−5.0 [22].

### Phylogenetic analysis and average nucleotide identity (ANI) calculation

To perform a comparative analysis, 28 most closely related species and representative SFGRs were retrieved from the NCBI (Table 1). Rickettsial representative markers, *groEL*, *gltA*, *17-kD* genes, and 16S rRNA gene sequences, and whole genomes were used to construct phylogenetic trees. Firstly, before the B8 genome sequenced, the sequences of *groEL*, *gltA*, *17-kD*, and 16S rRNA marker genes were obtained by paired primers amplification. Next, all amplicons were cleaned via Qiagen QIAamp mini spin columns (Qiagen Inc., Valencia, CA) and the amplification products were sent to GENERAL BIOL (Anhui, China) for Sanger sequencing (3730 DNA ANALYZER) (Table S1) [23]. In addition, the *groEL*, *gltA*, *17*-*kD* genes, and the 16S rRNA gene of other strains were extracted from the genomic sequences by using blast and Barrnap [24], respectively. The phylogenetic tree of *Rickettsia* spp. based on the above-mentioned marker gene sequences was constructed using the NJ method in MEGA v10.0 with 500 bootstraps replications [25]. However, phylogeny based on only one common gene would result in bias; therefore, we performed the phylogenetic analysis based on the concatenated sequences of *groEL*, *gltA*, *17-kD*, and 16S rRNA gene sequences. The multiple sequence alignments of single-copy genes were performed using MUSCLE and the phylogenetic tree was generated using the NJ method in MEGA v10.0 with 500 bootstraps replications. Furthermore, the phylogenetic tree based on core genome of incorporated *Rickettsia* strains was also constructed in our study according to previous [26,27]. In brief, multiple sequence alignments for these genomes were created using MUSCLE. The resulting alignments were used for phylogenetic analysis by employing Maximum-Likelihood (ML) with 100 bootstrap replicates. Evolutionary distances for ML were calculated leveraging Jukes-Cantor model [28] and visualized by EvolView [29]. The average nucleotide identity (ANI) values between the newly sequenced B8 genome and the representative genomes of other 28 public SFGRs were calculated using fastANI v1.2 based on a BLAST algorithm [30].

### Comparative genomics analyses

Whole-genome sequences of 29 *Rickettsia* strains were obtained for pan-genome analysis. Among which, 28 were publicly retrieved from National Center for Biotechnology Information (NCBI) database (Table 1). We leveraged Bacterial Pan Genome Analysis tool (BPGA) pipeline (v1.3) with default parameters for pan-/core-genome analysis [31], and all of orthologous among 29 *Rickettsia* spp. in this study were identified. The pan genome is referred to the whole set of genes in the test genomes. However, the core genome is the set of shared genes that is present in all tested strains. In addition, the accessory genome is the set of genes shared with more than two but not all tested strains. Furthermore, the unique genes are the set of genes in each strain not shared with other strains [32]. The details of the strains were showed in Table 1. A structural comparison via multiple genome alignment was performed using Mauve software [33]. Motif searches, for detecting conserved or variable motifs were performed using MEME [34], and the phylogenetic tree, conserved motifs were visualized using Tbtools software [35]. Gene sequences alignment and polymorphic sites identification were performed and visualized in JalView [36].

### Anti-*Rickettsia japonica OmpB* polyclonal antibody production

In this study, the anti-*Rickettsia japonica OmpB* polyclonal antibody was generated according to the previous report [37]. In brief, the sequence in the C-terminal of *OmpB* (*OmpBC*, 480 bp), was amplified by PCR from the genomic DNA of *Rickettsia japonica* deposited in our lab. Then, *OmpBC* was cloned into the pET28a plasmid with an N-terminal 6×His tag. Production of 6×His-*OmpBC* protein was induced in *Escherichia coli* str. BL21 (DE3) by the addition of 0.1 mM isopropyl-β-d-thio-galactoside (IPTG) for 4 h at 37°C. Bacterial cells were harvested and resuspended in lysis buffer (50 mM NaH_2_PO_4_, pH 8.0, 300 mM NaCl, and 10 mM imidazole) and stored at −80°C. For protein purification, the bacteria were thawed, lysozyme was added to 1 mg ml^−1^ (Sigma, L4919) and lysis was carried out by sonication. The lysate containing 6×His-*OmpBC* was incubated with Ni-NTA resin (Qiagen) and the bound proteins were eluted with 300 mM imidazole (in lysis buffer). The fractions were analysed by SDS-PAGE and those with the highest concentrations of 6×His-OmpBC were pooled before desalting, using PD-10 desalting columns (GE Healthcare, cat. no. 17085101) and equilibrated with lysis buffer lacking imidazole. Next, the *OmpBC* protein was fully emulsified with an equal volume of freund’s complete adjuvant at a dose of 0.8 mg/each by subcutaneous injection twice of Beijing white rabbits at two-week intervals. Finally, the polyclonal antibody was generated by the isolation of rabbits’ serum and the titre was determined by indirect ELISA.

### Immunofluorescence analysis and electron microscopy

Human microvascular endothelial cell (HMEC-1) cells were cultured in 12-well dishes containing coverslips coated with poly-L-lysine. The cells were fixed for 15 min on ice in 4% formaldehyde in PBS, followed by incubation with 0.1% Triton X-100 and then incubated for 2 h with primary antibodies anti-*Rickettsia japonica ompB*. After washing with 1× PBS for three times, cells were incubated for 1 h with Cy3 conjugated secondary antibodies (goat anti-Rabbit IgG, Beyotime) at 1:1000 in PBS containing 5% horse serum and 0.1% Triton X-100. DNA was stained with 0.5 µg/mL DAPI. Confocal images were acquired with LSM880 microscope (ZEISS) equipped with 60× Plan Apochromat VC objective and airyscan detector. For the transmission electron microscopy, rickettsia-infected HMEC in each time point were harvested by centrifugation and fixed using 2.5% glutaraldehyde for 3 h, followed by washing with 1× PBS for three times. The next step in sample preparation before imaging is post-staining. Typically, the sections were stained in 2% aqueous uranyl acetate for 20 min, washed well with distilled water, followed by staining in Reynold’s lead citrate for 15 min, and washed again with distilled water to enhance the contrast [38]. After air-drying, TEM images of the specimens were captured with Tecnai G2 transmission electron microscope at an accelerating voltage of 120 kV.

### Ethical statement

Only patient serum-isolated bacteria were used in this study. No human subjects participated in this study. Animals used in this work were approved by the ethics committee of Anhui Medical University Biomedical Ethics Committee (Approval No. 2021H021).

## Results

### Identification of B8 by clinical presentation, immunofluorescent, and morphological characterization

On 17 April 2020, a patient was hospitalized and complained of a febrile illness with fever at 39.0° and other clinical signs, including asthenia, anorexia, nausea, and skin rashes all over the body. The patient confirmed that she had been bitten by ticks under the armpit five days ago when picking tea on a mountain. Physical examination identified a 1.3 × 1.1 cm^2^ erythematous rashes around the tick-bite eschar, and vesicles were seen surrounding the bite site. A papular rash with papules ∼0.1–0.3 cm in diameter was found all over the body. Blood samples were collected, and routine laboratory and haematologic assays were performed. Because of the rashes found during physical examination, infection with a spotted fever group *Rickettsia* was suspected. Therefore, the patient was given doxycycline on the day of admission. The patient was successfully cured and discharged in three days.

**Figure 1.**
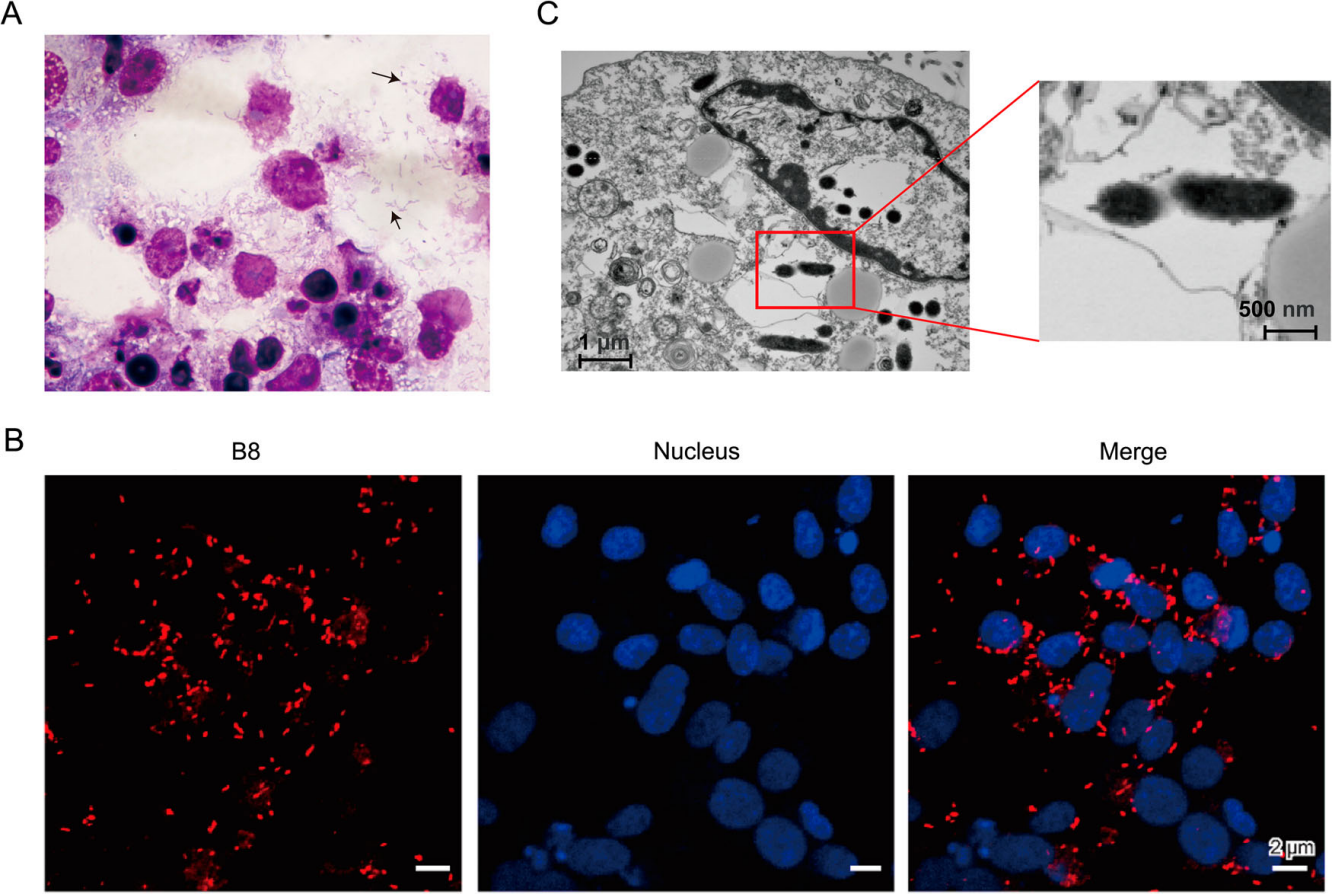
Morphological and biochemical characterization of *Rickettsia heilongjiangensis* strain B8. (A) Diff-Quick stained smears of Vero cells showed Rickettsia-like bacilli in the cytoplasm. The black arrow points to an individual bacterium. (B) Immunofluorescence microscopy of B8-infected Vero cells. DAPI (blue) was used to stain host cell nucleus; polyclonal anti-*ompB* antibody followed by Cy3 conjugated secondary antibody (red) was used to stain B8. (C) Scanning electron microscope (SEM) micrograph showing numerous Rickettsia-like organisms parasitizing in the cytoplasm of infected Vero cells.

Afterwards, the patient’s blood specimens collected at the first day of admission were inoculated onto THP-1 and Vero cells. Cytopathic effects were observed for both cell lines after two weeks of cultivation. Firstly, the Diff-Quick stained smears of Vero cells showed *Rickettsia*-like bacilli in the cytoplasm (Figure 1(A)). Then, Immuno-fluorescence assays were then performed using a polyclonal antibody against *Rickettsia japonica ompB* every two days, which exhibited rod-shaped particles in the inoculated cultures (Figure 1(B)). In addition, transmission electron microscopy revealed a typical ultrastructure of *Rickettsia* bacteria with dimensions of approximately 0.2 μm × 1.0 μm (Figure 1(C)).

### Local epidemic zone investigation of *R. heilongjiangensis* and taxonomic, genomic characterization of B8

Firstly, to identify the local natural foci, adult ticks, including 12 *Ixodes persulcatus*, 175 *Haemaphysalis longicornis*, and 21 *Dermacentor silvarum*, were collected in vegetation near where the patient lived (Figure S1(A)). DNA was extracted from these ticks. The *17-kDa*, *16S rRNA*, *groEL*, and *gltA* genes were amplified using the corresponding primers (Table S1). The specificity of these primers was examined by agarose gel electrophoresis of the PCR product (Figure S1(B)). As a result, *R. heilongjiangensis* was identified in 11 of 175 *H. longicornis* ticks and 0 of the other ticks tested (Table S2).

**Figure 2.**
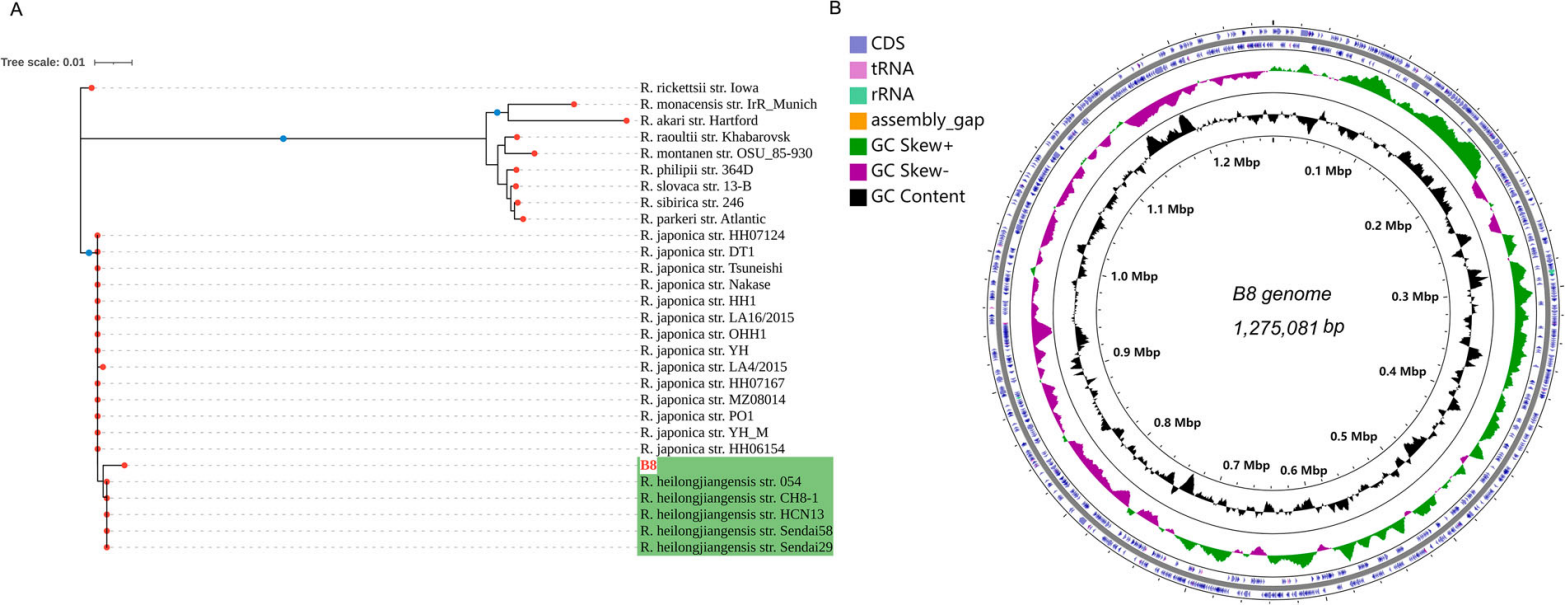
Genome structures and phylogenetic relatedness of B8. (A) Phylogenetic tree (Neighbour-joining) construction based on the concatenated multiple-sequence alignment (MSA) of four representative genes (*gltA*, *groEL*, *17-kD*, and 16S rDNA). B8 highlighted in red was clustered with five other *R. heilongjiangensis* strains with green background. (B) Circular genome maps of B8. The four circles (outer to inner) show the following. Circle 1 and 2 exhibit CDS on the forward and reverse strands, as well as rRNA, tRNA, and assembly gap. Circle 3 represents the GC skew ((C − G)/(C + G)) curve (positive GC skew, dark green; negative GC skew, violet). Circle 4 shows the GC content.

**Figure 3.**
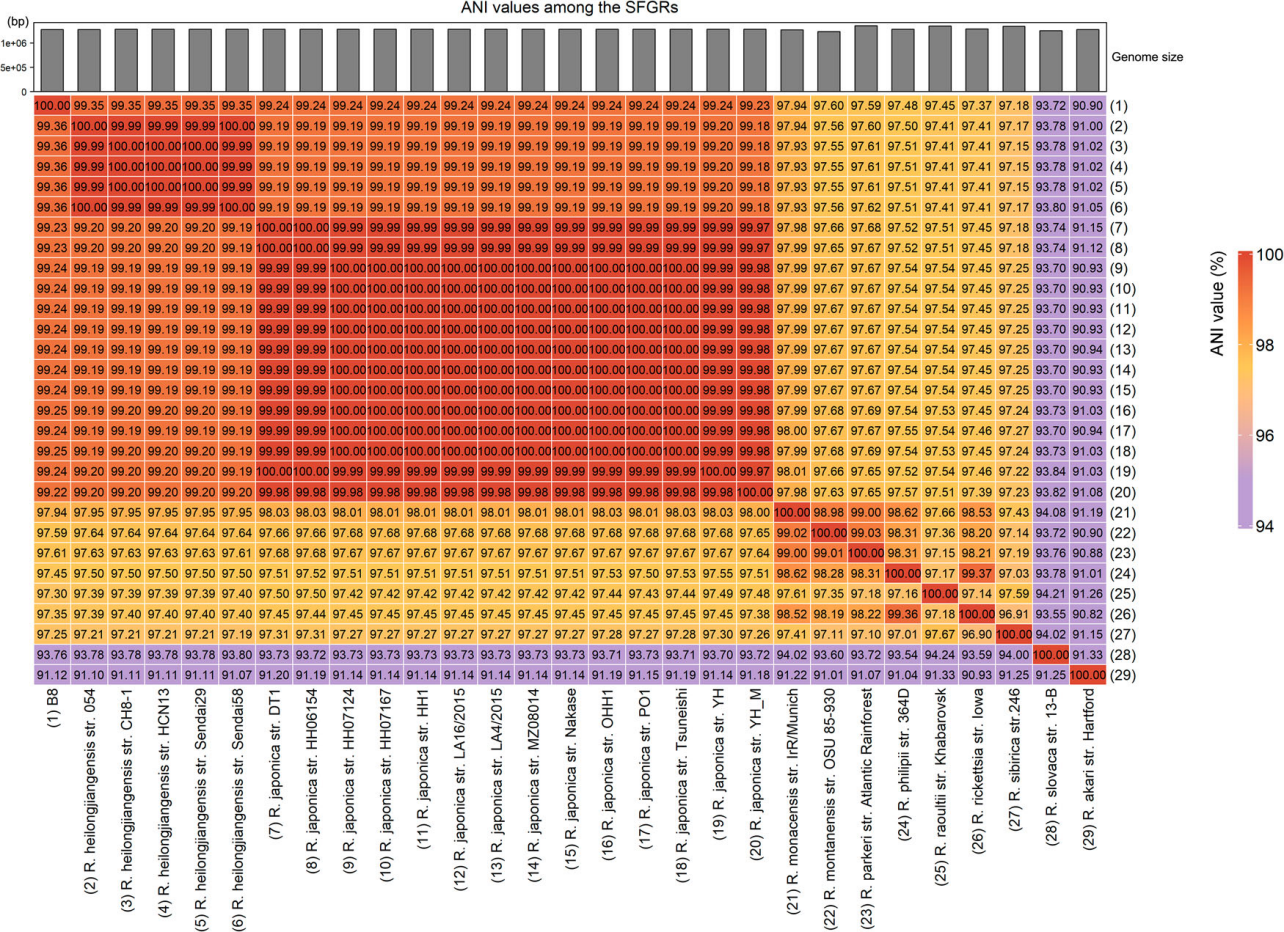
Heat-map of Average Nucleotide identity (ANI) values amongst different lineages representative strains of SFGR and B8. The bar in the head of heat-map shows the size of genome.

Subsequently, DNA was extracted from purified bacteria propagated by culturing in Vero cells. Based on the immunofluorescence results, we tentatively determined that B8 could be a new strain of *Rickettsia japonica* or another SFGR strain. Firstly, we amplified several marker genes, such as *17-kDa*, *16S rRNA*, *groEL*, and *gltA* with specific primers (Table S1), and then delivered to sanger sequencing. We concatenated the sequences of these genes and constructed a phylogenetic tree, shown that the amplified sequences from B8 clustered into the same clade as *R. heilongjiangensis* (Figure 2(A)). Next, WGS of B8 yields a total of 2,038,576 quality-filtered reads, 604 Mb of sequence data. After subjected to de novo assembly, we found the draft genome of B8 consists of 82 contigs ranging in size from 1108 to 11,183 bases, resulting in a circular chromosome of 1,275,081 bp, with an overall 32.4% GC content. A total of 1441 coding sequences (CDS) were predicted to be protein coding (Figure 2(B), Table S3). Based on the whole-genome annotation against bacterial RefSeq database by Kraken software, B8 was verified as belonging to *R. heilongjiangensis*. This was further corroborated with the phylogenetic analysis based on the WGS using core-genome (Figure S1(C)). According to the results obtained from WGS-ANI (average nucleotide identity) analysis using fastANI, the genome of B8 was nearly identical to *R. heilongjiangensis* and *R. japonica* clusters, and the values of ANI between each two strains was more than 99.35% and 99.22%, respectively (Figure 3).

However, none plasmids were detected in the B8 genome, which is consistent with the genomic feature of type strain *R. heilongjiangensis* 054 [8], and also no IS elements were identified. In the present study, however, two incomplete prophage regions covering a sum of 5 most likely prophage-like proteins were discovered in the genome of B8 (Figure S2, Table S4). Accumulating evidences suggest that the surface proteins of rickettsial organisms play a crucial role in mediating the direct *Rickettsia*-host interaction. Thus, we profiled the putative surface proteome of the B8 strain using Signal-P (v5.0) which detects cell membrane (Table S5). Altogether, 63 genes were predicted as signal peptide secreted in general secretory pathway and cleaved by signal peptidase I (Sec/SPI), and 35 genes were predicted to be associated with lipoproteins and cleaved by signal peptidase II (Sec/SPII) annotated.

### Unravelling the gene function in the genome of *R. heilongjiangensis* strain B8

**Figure 4.**
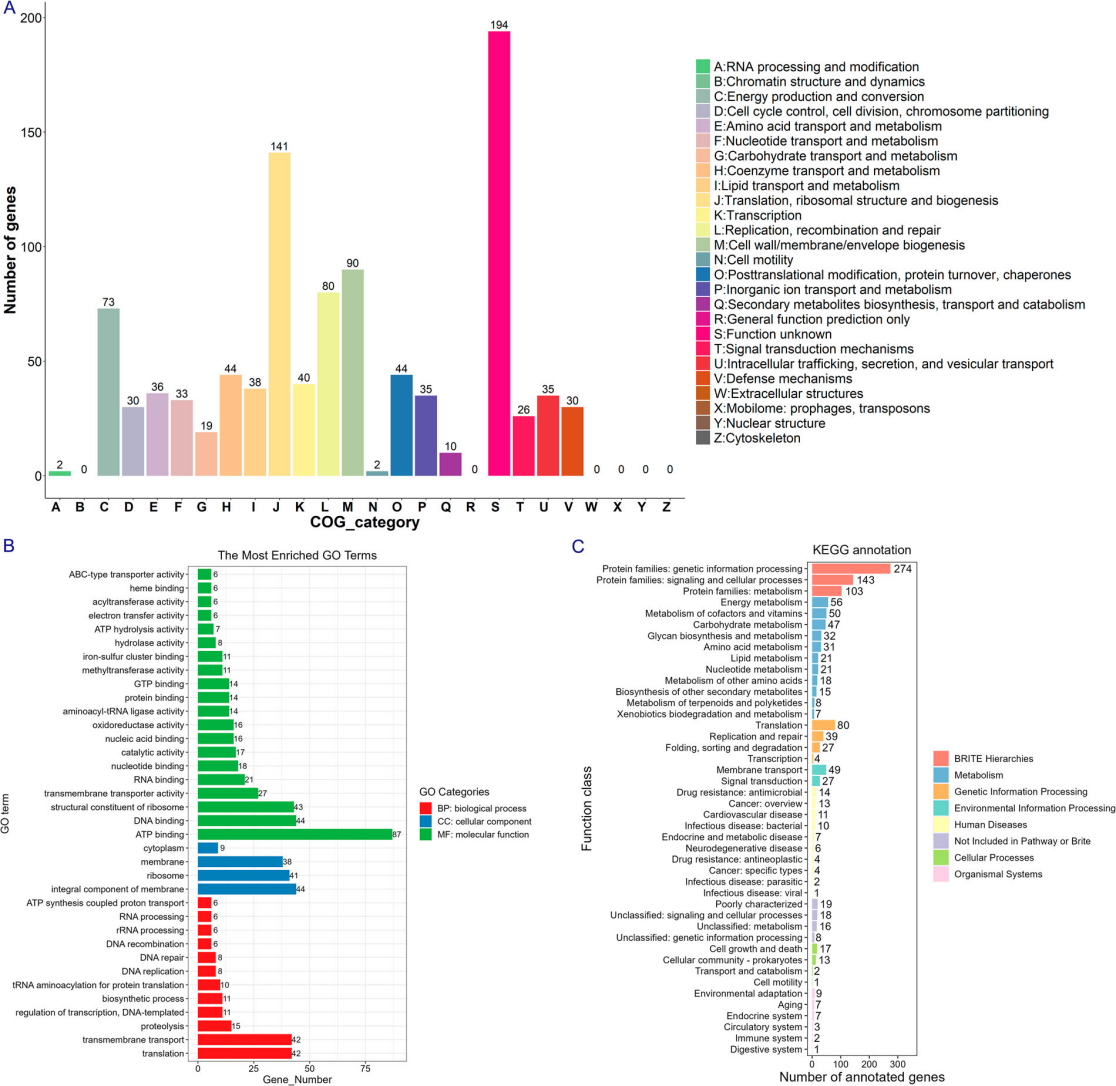
Functional category analysis of the B8 genome annotated to the COG, GO, and KEGG databases. (A) COG annotation assignments. The COG functional annotations were divided into 26 categories. The COG categories are shown on the X-axis as alphabets, with category names on the right. (B) GO annotation distribution (level 2). The GO assignments were divided into three categories (level 1) namely, biological process (red), cellular process (blue), and molecular function (green). (C) KEGG annotation distribution. The KEGG orthologies were categorized into eight major categories: Brite Hierarchchies, Metabolism, Genetic Information Processing, Environmental Information Processing, Human Diseases, Not Included in Pathway or Brite, Cellular Processes and Organismal Systems.

The genes of B8 showed a wide range of functional categories when annotated to COG, GO, and KEGG databases, mainly encompassing the categories representing cellular process, metabolic process, and catalytic activity (Figure 4(A–C)). In addition, according to the annotation results of CAZy, we found that the B8 genome contains five major families of glycoside hydrolase (GH), glycosyl transferase (GT), carbohydrate-binding enzyme (CBM), auxiliary active enzyme (AA), and polysaccharide lyase (PL), in which the GT family is the most abundant CAZy genes (Figure S3).

### Virulence factors determination

**Figure 5.**
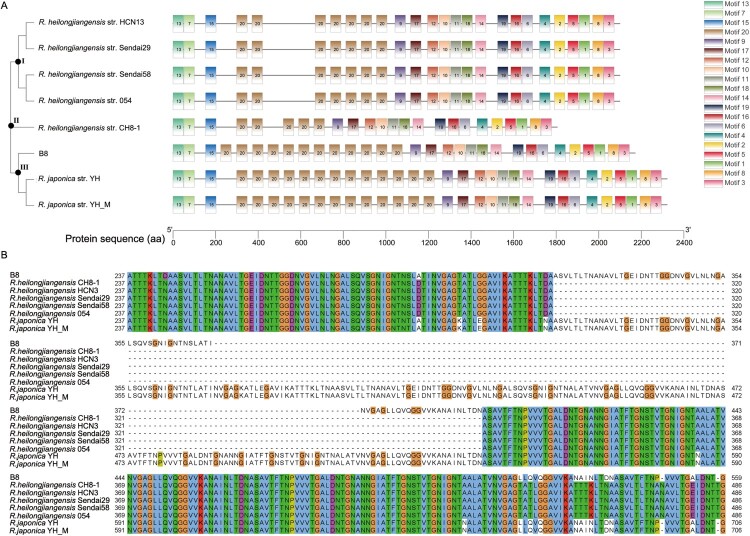
Analyses of *ompA* gene variations among B8, other 5 *R. heilongjiangensis* and 2 *R. japonica* strains. (A) Phylogenetic analysis of *ompA*, constructed by the neighbour-joining method in MEGA 10.0 software, and identification of conserved motif by MEME online tool. The tree and motifs distribution were visualized by TBtools. (B) For a portion of the polymorphic sites, showing sequence variation of *ompA* gene in the B8 genome among the selected SFGRs. The sequences were aligned with MUSCLE algorithm and visualized with Jalview.

In terms of VFDB analysis, 30 predicted genes in the genome of B8 were aligned with 30 virulence genes using *R. heilongjiangensis* 054 as reference. The virulence genes could be divided into four classes, in which the Actin-based motility (*RickA*, *Sca2*), adherence and invasion (*rOmpA/Sca0*, *rOmpB/Sca5*), enzyme and secretion system (T4SS) were presented in all strains of representative *Rickettsia* spp. Particularly, we found phospholipase A2 (*pat2*) was specific in the B8 genome in comparison with reference genome of *R. heilongjiangensis* strain 054 (Table S6). In order to reveal the existence of difference in virulent gene structure within *R. heilongjiangensis* strains, the predicted *ompA* gene was selected as representative for motif search and phylogenetic analysis by the N-J method with 500 bootstrap replicates. Twenty motifs were identified with duplicates in terms of *ompA* gene structure. Interestingly, the tree is divided into three clades (clade I, II and, III). Clade I involves four strains within *R. heilongjiangensis* (Sendai58, 054, Sendai29, and HCN13), clade III contains *R. heilongjiangensis* strain B8 and 2 strains within *R. japonica* (YH and YH_M), and *R. heilongjiangensis* strain CH8-1 belonging to clade II (Figure 5(A)). These differences are mostly single nucleotide variation and verified by the amino acid sequences alignment with MUSCLE using Jalview tool (Figure 5(B)). These results revealed that there are several disparities in the genome of B8 compared to its intra- or interspecies *Rickettsia*.

### Multiple comparative genomic analysis across the B8 and other SFG *Rickettsia*

To expand the insight into the difference in genomic composition and structure of B8 across other inter-/intra-SFGR species, a comparative genome analysis was performed to find orthologs among the genome of B8 and additional 28 available but completed SFGR genomes from NCBI. In total, we found 5,117 orthologs in the pan-genome of 29 SFGRs, containing 254 core genes and 4863 (95.03%) accessory genes (Figure S4(A)). Notably, the pan-genome experienced a dramatic increase when strains of *R. akari*, *R. monacensis*, *R. montanen*, *R. parkeri*, *R. philipii*, *R. raoultii*, *R. sibirica*, and *R. slovaca* were taken into analysis (Figure S4(B)). Furthermore, multiple genome alignment demonstrated the consistency or discordance among the B8, *R. heilongjiangensis*, *R. japonica*, and other SFGRs using the Mauve tool (Figure S5).

**Figure 6.**
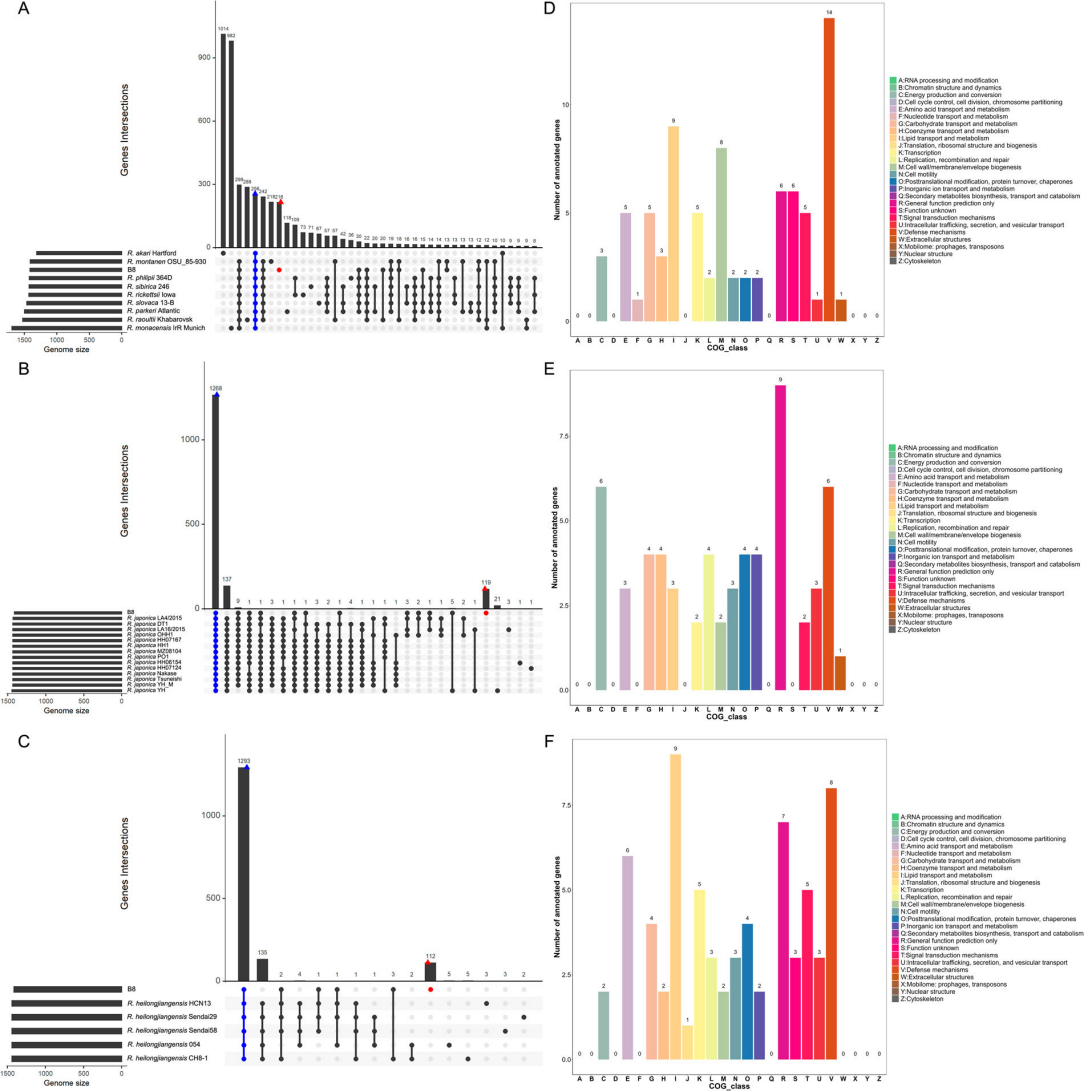
Genomic comparison amongst the B8 and additional SFGRs. (A–C) Upset diagram illustrating the number of core genome, shared orthologous and unique genes among B8 and 9 other SFGRs, B8 and other 14 *R. japonica*, as well as B8 and other 5 *R. heilongjiangensis* based on complete genome sequence of the selected strains in the present study. Vertical barplot indicates the number of overlapping genes (intersection size) for each intersection represented by the connected dots. The grey horizontal barplot, reported as “Gene Number,” indicate the total number of genes in each Strain. (D–F) COG annotation and categories assignment based on 216, 119, and 112 B8-unique genes.

An open pan-genome could show the diverse response of the different taxa of *Rickettsia* to divergent environments. To gain a better understanding of the functional features of the pan-genome and identified specific genomic signature in the genome of B8. Comparative genomic analysis among the B8 and other nine SFGRs (not including 5 *R. heilongjiangensis* and 14 *R. japonica* strains) showed that a sum of 216 unique genes was found in the B8 genome (Figure 6(A)). In addition, 119 and 112 genes were found exclusively in the genome of B8 when compared with 14 *R. japonica* and 5 *R. heilongjiangensis* strains (Figure 6(B,C)). Collectively, COG annotations revealed that those B8-specific genes were mainly assigned to “[V] defense mechanism”, “[I] lipid transport and metabolism,” “[M] cell wall/membrane/envelope biogenesis,” “[C] Energy production and conversion,” and “[E] amino acid transport and metabolism” (Figure 6(D–F)).

Next, the phylogenetic relationships among B8, *R. heilongjiangensis* strain 054, *R. japonica* str. YH_M and nine other SFGRs were investigated. We found these 12 strains appear to have 255 ortho-groups in common and 36 specific homologous genes in the B8 genome (Figure S6(A)). However, the total number of specific genes in the B8 genome was less than in the genome of *R. heilongjiangensis* 054 (*n* = 58) and *Rickettsia japonica* YH_M (*n* = 43), though the taxonomy of B8 was classified as *R. heilongjiangensis*. Specifically, those 36 B8-specific genes were also predominantly classified into “[V] defense mechanisms” and “[I] lipid transport and metabolism” (Figure S6(B)).

## Discussion

In recent years, the variety and distribution of SFGR in China have been showing a trend of diversification [39–42]. Therefore, the precise identification and differentiation of SFGR and the elucidation of molecular mechanisms and pathogenesis are the focus of current research. It is well recognized *R. heilongjiangensis* 054 was first isolated from *D. silvarum* ticks in the Heilongjiang province, China. In this paper, we isolated a new strain of *R. heilongjiangensis* in Anhui Province, the central-eastern part of China, which suggests the epidemic zone of *R. heilongjiangensis* has been expanded or its occurrence has been detected in new regions. In addition, we confirmed the presence of *R. heilongjiangensis* in *H. longicornis* ticks collected near where the patient, from which the B8 strain was isolated, lived. This is a potentially new vector for *R. heilongjiangensis* and warrants further investigation. *Haemaphysalis longicornis* is reported widely distributed in China and around the world [43], suggesting that we need to concentrate on the prevention and the control of the spread of *R. heilongjiangensis* in other tick hosts. In regarding to the WGS of B8, we first classified the isolate as a new strain of *R. heilongjiangensis* according to the reliable taxonomic annotation and precise phylogenetic analysis based on the core-genes of WGS, as well as morphological and immunofluorescence examination. We then deciphered the genomic architecture and functional information by complete genome sequencing. In terms of the genomic composition of B8, a total of 1411 CDSs were annotated. Next, we identified 1293 genes from the core genome of B8 and other five publicly *R. heilongjiangensis* strains from NCBI. Additionally, we noticed there were 112 unique genes in the B8 genome, which suggested the existence of genetic differences compared to other *R. heilongjiangensis* species. Overall, the present research revealed several divergences in the genomic composition of B8 as compared with other publicly *R. heilongjiangensis* and *R. japonica* strains. We thought B8 rather represents a previously undescribed human-pathogenic SFG rickettsia lineage, may be an intermediate of these lineages. Hence, the results suggested that we should gain a deep understanding of the genetic background of the *R. heilongjiangensis* strains before we know their biological potential, including their pathogenicity for humans and symbiotic relationship with the host, clarification of the metabolic pathways associated with intracellular parasitism.

The Majority of spotted fever group rickettsiae are transmitted to humans via a tick bite. Even if some *Rickettsia* species, such as *R. rickettsii*, are deleterious to host ticks infected by transovarial transmission in nature, a majority are known to persist and survive in infected vectors and lead to serious disease in humans and vertebrates [44–46]. We found that the genome of *R. heilongjiangensis* strain B8 contains the common virulence factors which are conserved among SFG rickettsiae, suggesting a major role of the virulence genes in determining its pathogenicity. Previously reports have demonstrated the pivotal role of *ompA* (*Sca0*) gene in mediating the attachment and invasion of SFG rickettsiae, however, partially dependent on a conserved non-continuous RGD motif present in a predicted C-terminal “pertactin” domain in *ompA* [47–49]. In this research, phylogenetic analyses, motif identification, and polymorphic sites investigation based on *ompA* gene sequence in the genome of B8 and other SFGRs showed obvious divergence, we concluded that there was minor gene flux between interspecies of *R. heilongjiangensis* and *R. japonica*. In addition, we found the virulent gene *pat2* in the B8 genome, belonging to phospholipase A2, which was inconsistent with the type strains *R. heilongjiangensis* 054 and *R. japonica* YH. In light of previous research, rickettsial phospholipase A2 has been reported to mediate the entry into the host cell, escape from the phagosome, and cause damage to host cells via both typhus and spotted fever group rickettsiae [50,51]. These results together revealed the phylogenetic close relationships among the B8 and other *R. heilongjiangensis* strains, nevertheless, B8 also has different evolutionary patterns because of the two representative virulent genes, *ompA* and *pat2*. In addition, those B8-specific genes obtained from the pan-genome analysis offered an obvious hint that the genome evolution of B8 might go with the divergent geographical location and different tick hosts that reside in. Collectively, the genomic message of *R. heilongjiangensis* B8 delivered valuable information regarding the molecular function, pathogenicity, and genomic divergence when compared with the inter/intra-species *Rickettsia*. In addition, the variants of several genes, such as *ompA* and *pat2*, combined with a bulk of genes exclusive to B8 offered a substantial range of genetic markers for the detection and surveillance of new pathogenic *R. heilongjiangensis*, and also provides clues for subsequent studies on the bacterial pathogenicity.

Overall, the present study confirms the transmission of spotted fever *R. heilongjiangensis* with pathogenicity in East-Central China for the first time. This further suggests that *H. longicornis* might be a vector of *R. heilongjiangensis* or harbours it, which may broaden the variety of ticks as carrier of *R. heilongjiangensis*. As such, caution should be taken in districts with a distribution of the *H. longicornis* to avoid the infection of humans and animals by spotted fever *R. heilongjiangensis*. However, whether these B8-unique genes are associated with virulence and pathogenesis warranting further investigation.

## Supplementary Material

Supplemental Material

## Data Availability

The complete genome sequence of *Rickettsia heilongjiangensis* strain B8 was deposited at the NCBI’s GenBank with the accession ID: CP112971.
