# Supplementary figures and images for: Complete genome sequencing and comparative genomic analyses of a new spotted-fever Rickettsia heilongjiangensis strain B8
Source: Emerg Microbes Infect. 2023 Feb 13;12(1):2153085. doi: 10.1080/22221751.2022.2153085 (PMC9930820; doi:10.1080/22221751.2022.2153085)

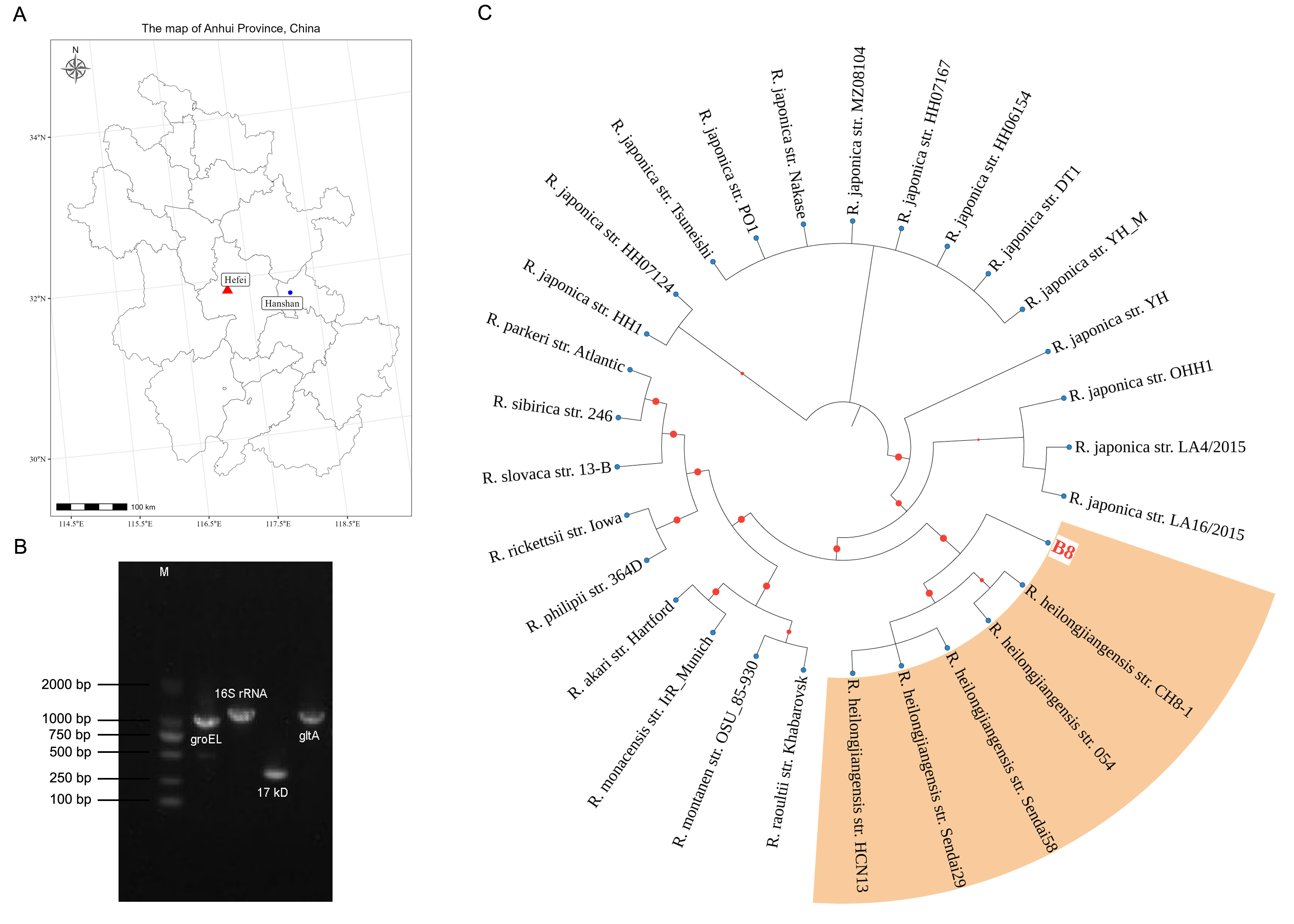

Supplement: Supplemental Material [file TEMI_A_2153085_SM4731.zip › Figure S1.tif]

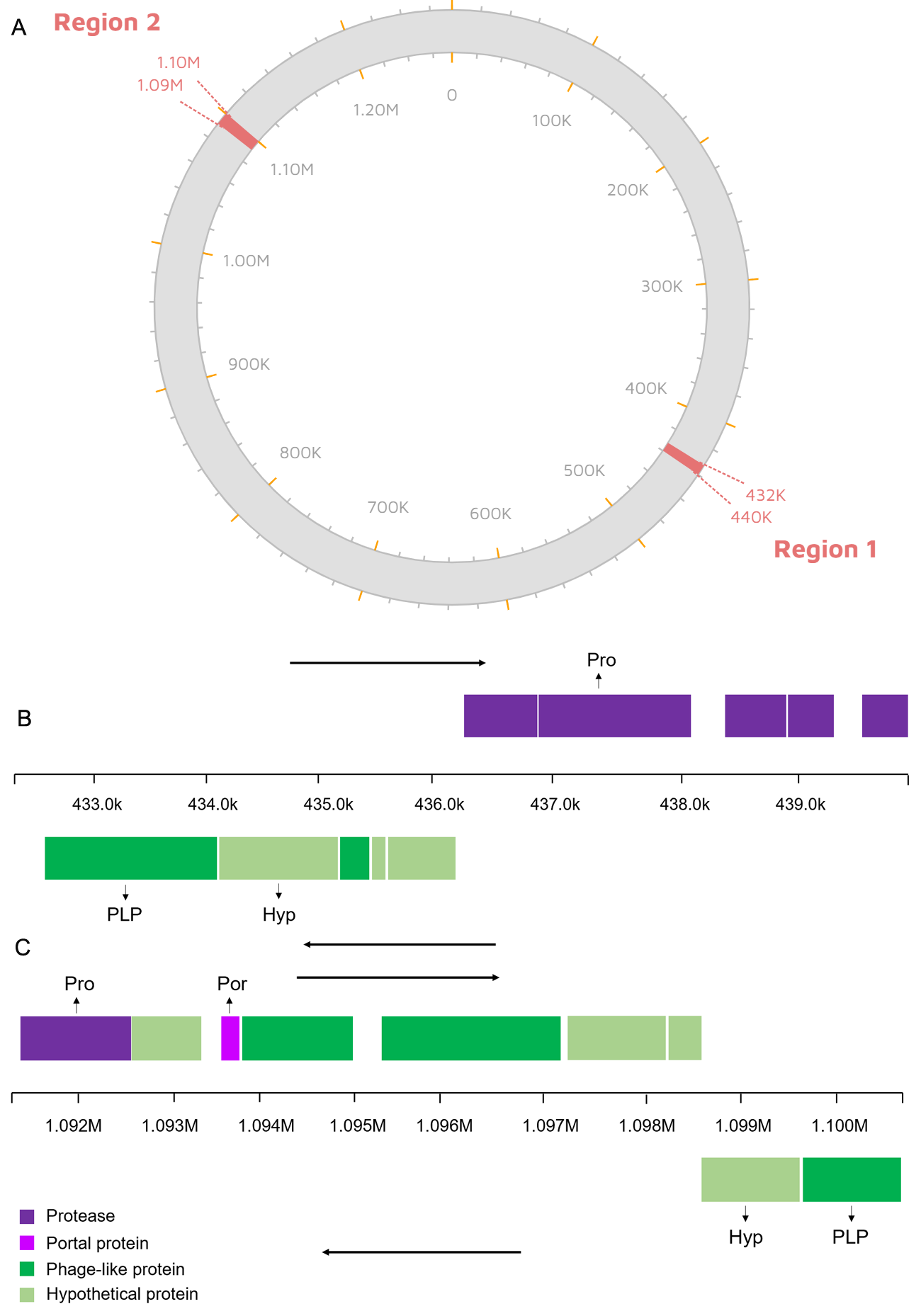

Supplement: Supplemental Material [file TEMI_A_2153085_SM4731.zip › Figure S2.tif]

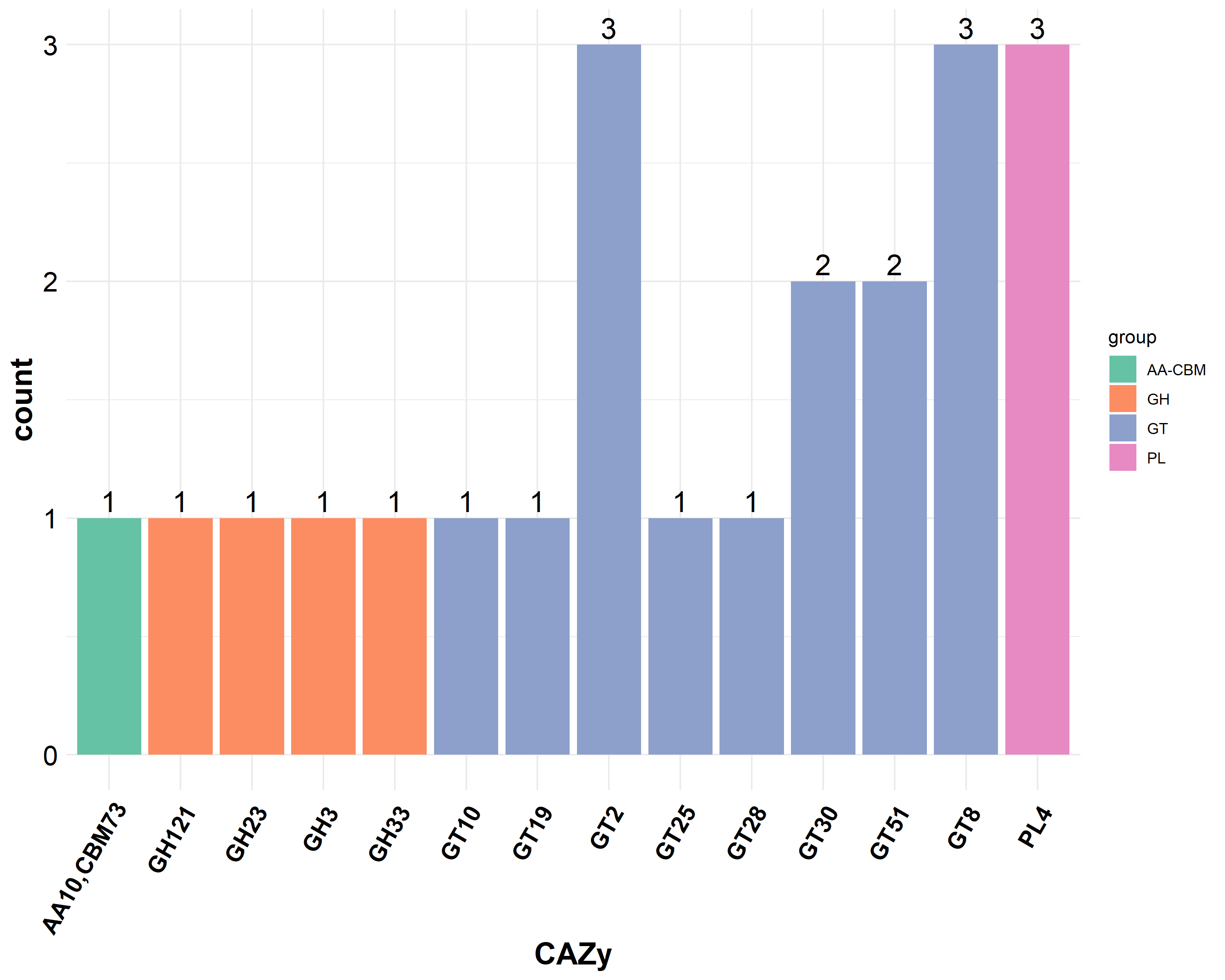

Supplement: Supplemental Material [file TEMI_A_2153085_SM4731.zip › Figure S3.tiff]

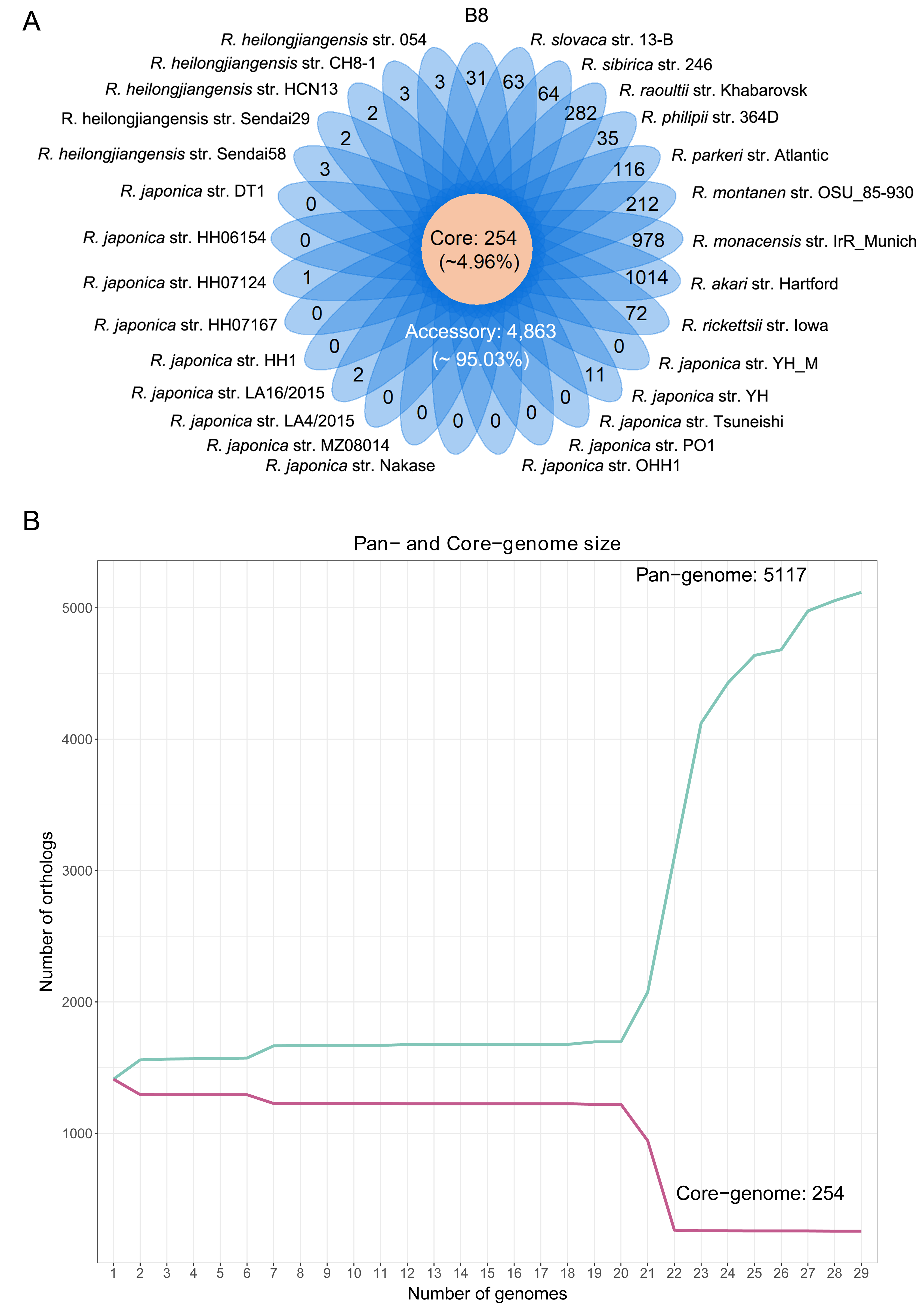

Supplement: Supplemental Material [file TEMI_A_2153085_SM4731.zip › Figure S4.tiff]

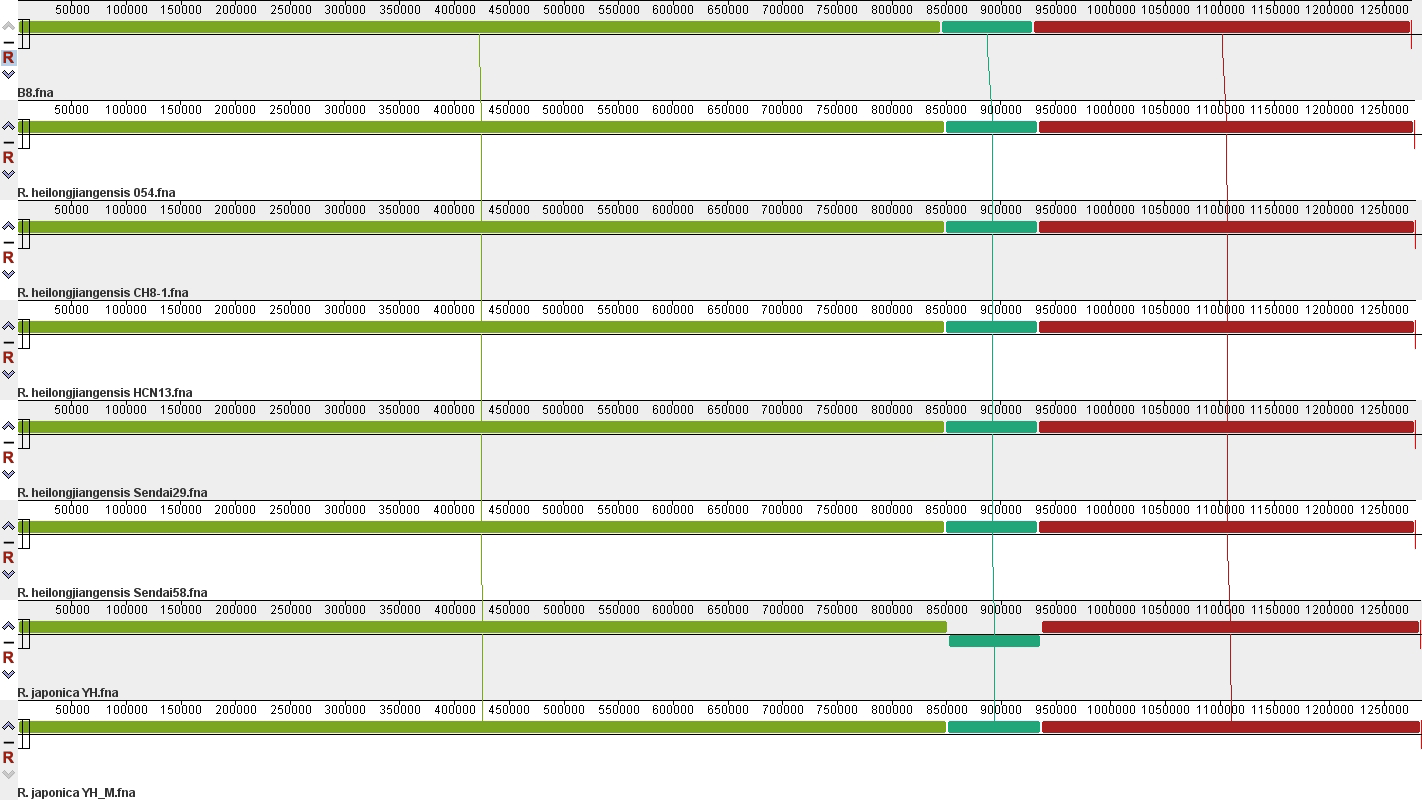

Supplement: Supplemental Material [file TEMI_A_2153085_SM4731.zip › Figure S5.tiff]

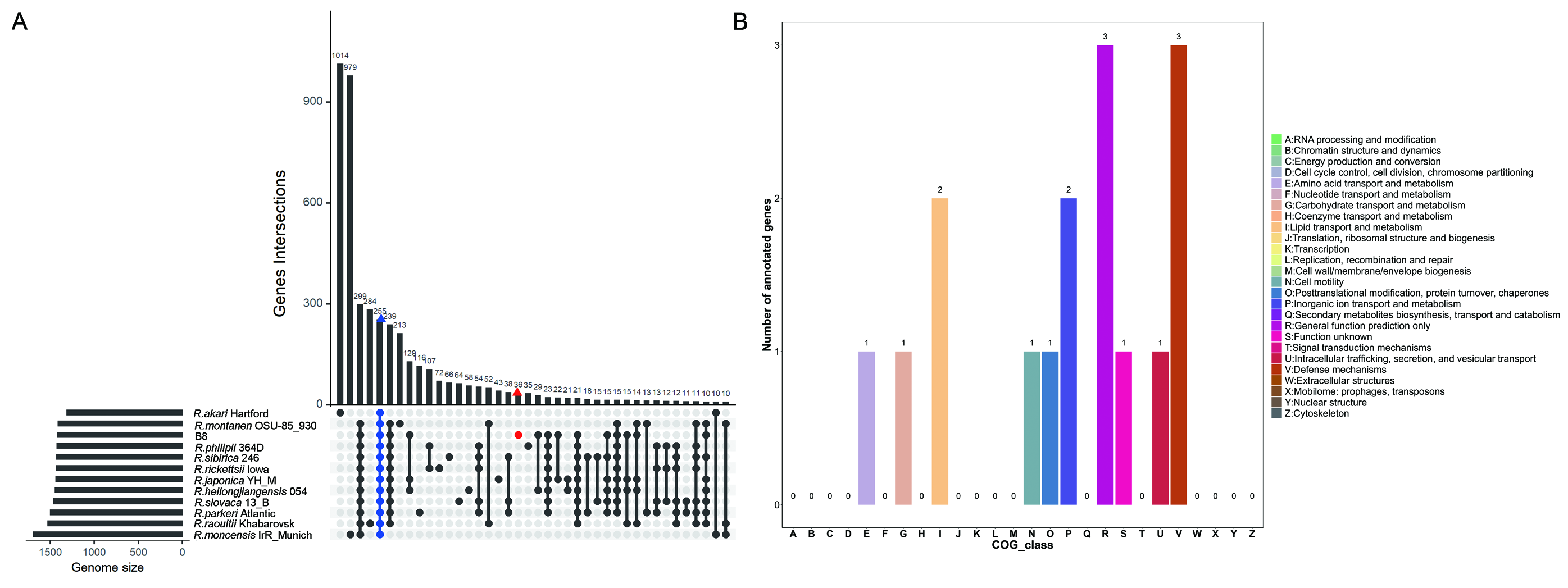

Supplement: Supplemental Material [file TEMI_A_2153085_SM4731.zip › Figure S6.tiff]
